# Commentary: Musculoskeletal adverse events in dogs receiving bedinvetmab (Librela)

**DOI:** 10.3389/fvets.2025.1649240

**Published:** 2025-12-15

**Authors:** Alfie Lloyd

**Affiliations:** Academic Unit of Anaesthesia, Critical Care and Perioperative Medicine, University of Glasgow, Glasgow, United Kingdom

**Keywords:** pharmacovigilance, evidence-based medicine, statistical accuracy, disproportionality analysis, osteoarthritis, dogs, canine osteoarthritis

Unfortunately, the recent article “Musculoskeletal adverse events in dogs receiving bedinvetmab (Librela),” by Farrell et al., in Frontiers of Veterinary Science is deeply flawed (1). The results of their pharmacovigilance analysis must be discarded entirely due to key methodological errors, and the conclusions of the case series must be caveated for excluding dogs not treated with Librela. Perhaps more worryingly, the manuscript contains several gross errors and misstatements. Given how much attention this article has received (>230,000 views at the time of submission) and the punchy conclusions reached by the authors, highlighting these issues swiftly is paramount.

## Disproportionality analysis

There are two major issues with the pharmacovigilance analysis. Firstly, the authors fail to actually perform a disproportionality analysis, rendering their results uninterpretable. Disproportionality analysis aims to offset a fundamental limitation of conducting pharmacovigilance using adverse drug reaction (ADR) reports: the databases do not include the total number of treated animals. Without the total, one cannot know the proportion of animals that developed the ADR. It also makes comparing drugs difficult, since a safe drug used extensively may have more reports than a dangerous one taken rarely. Take the ADR reports of paracetamol and meloxicam for humans (2, 3). There were 7200% more ADRs reported for paracetamol than meloxicam in 2024 (727 vs. 10). Is it likely that ADRs occur ~72 times more frequently with paracetamol compared to meloxicam, or might the result just represent the fact that paracetamol is taken more frequently?

Disproportionality Analysis is a simple methodology for offsetting this problem. When looking at a specific ADR, two drugs can be compared using the proportional reporting ratio (PRR). “The proportional reporting ratio (PRR) is defined as the ratio between the frequency with which a specific adverse event is reported for the drug of interest (relative to all adverse events reported for the drug) and the frequency with which the same adverse event is reported for all drugs in the comparison group (relative to all adverse events for drugs in the comparison group)” (4). The PRR uses the total number of ADRs as a rough surrogate for the total number of doses. This PRR methodology is the basis of the FDA's analysis of Librela, and while Farrell et al. describe their methodology as performing a disproportionality analysis, they do not. Instead, their results are simply the raw number of specific ADRs. Using the ADR “Gastrointestinal disorders” (which include GI bleeding) from the above paracetamol/meloxicam example illustrates the problem. Paracetamol has 4,605 such reports out of a total of 30,439 ADRs or a reporting ratio of 15.1%. For meloxicam that ratio is 1,284 out of 3,009 for 42.7%. Therefore, the true PRR for meloxicam (42.7/15.2) is ~3 indicating that an ADR is 3 times more likely to be a GI disorder for meloxicam than paracetamol. But using Farrell et al.'s approach, the result is also around 3 but reversed (4,605/1,284), suggesting that GI ADRs are more likely for paracetamol than the NSAID meloxicam.

The second issue is the high risk of selection bias introduced by how the database search was conducted (5). A total of 79.5% of possible ADRs relating to MSK and neurological syndromes were filtered from the database. Given the data structure of the EudraVigilance database with the multiple overlapping lower-level terms (LLTs), this itself is not unreasonable. However, the filtering methodology becomes vital to prevent bias. The filtering methodology should be specified before seeing the data, and should be made available as supplementary material. Ideally, it would be done independently by multiple investigators with a procedure, such as a Delphi process, for adjudicating disagreements (6). Instead, filtering was done by a single author (Farrell) without any documentation of the criteria used.

## Case series

The methodology of the case-series component of the paper also makes the results problematic to interpret. The authors have tried to achieve two aims simultaneously: to characterize Rapidly-Progressive OsteoArthritis (RPOA) in dogs and associate Librela with RPOA. To do so, they limited their inclusion criteria to dogs who had received Librela. This has two consequences: first, it limits the number of cases from which to characterize RPOA. If it transpires that Librela is the sole causative agent of RPOA, they have the same number of cases and the ability to say that 100% were associated with Librela. Second, it makes drawing any conclusions about Librela's role in the disease impossible. As an analgesic, it is plausible that a clinician might prescribe Librela for a dog presenting at the early stages of RPOA. Therefore, even if it transpired that Librela was protective against developing RPOA, it is inevitable that there will be cases prescribed Librela that subsequently go on to be diagnosed with RPOA.

Beyond these methodological issues, but perhaps more disqualifying are some statements within the manuscript which suggest a worrying disregard for factuality and standards of scientific rigor. The most glaring example is describing ABON as standing for “Algorithm for Bayesian Onset of symptoms,” citing as evidence a paper which clearly states, “The ABON system categorizes adverse reactions as probable (A), possible (B), unlikely (O) or unclassifiable (N)” (7). The same reference is subsequently used to bolster their claim that “the FDA does not use sales-figure-based prevalence estimates, because they can dramatically underrepresent true incidence” despite neither the FDA nor any mention of drug sales appearing in the text. There is also an unreferenced claim that Bayesian analysis may have contributed to the opioid epidemic, which is almost too bizarre for comment.

I want to be clear: I am not defending Librela. The FDA study combined with the experience of anti-NGF drugs in humans and the limited pre-licensing RCTs in dogs is concerning. Furthermore, as a human clinician, I have no dog in this fight. But, as the authors note, the “media attention” and a “climate of apprehension and confusion” that surrounds Librela “requires unbiased and rigorous post-marketing pharmacovigilance to evaluate this drug's true risk-benefit profile.” This article does not provide such a profile but, as representatives of 15 separate institutions across 5 countries, they likely have the means to do so. A well-designed cohort study matching dogs presenting with symptoms of osteoarthritis who are started on different analgesic regimes and either surveying the owners for ADRs or simply tracking their outcomes over time would provide invaluable insights for clinicians and owners.
